# Factors associated with consumption of alcohol in older adults - a comparison between two cultures, China and Norway: the CLHLS and the HUNT-study

**DOI:** 10.1186/s12877-017-0562-9

**Published:** 2017-07-31

**Authors:** Juan Li, Bei Wu, Geir Selbæk, Steinar Krokstad, Anne-S. Helvik

**Affiliations:** 1 Nursing School of Second Military Medical University, Room 207 800 Xiangyin Road, Yangpu District, Shanghai, 200433 China; 2 Duke University School of Nursing, Durham, USA; 3 New York University Rory Meyers College of Nursing, New York, USA; 4 Shanghai University School of Sociology and Political Science, Shanghai, China; 5 The Norwegian Advisory unit for Aging and Health, Vestfold Health Trust, Tønsberg, Norway; 6 Center for Old Age Psychiatric Research, Innlandet Hospital Trust, Ottestad, Tønsberg, Norway; 7 Institute of Health and Society, Faculty of Medicine, University of Oslo, Tønsberg, Norway; 8 Department of Public Health and General Practice, Faculty of Medicine, Norwegian University of Science and Technology, Tønsberg, Norway; 9 HUNT Research Centre, Department of Public Health and General Practice, Faculty of Medicine, Norwegian University of Science and Technology (NTNU), Levanger, Norway; 10 Levanger Hospital, Nord-Trøndelag Health Trust, Levanger, Norway; 11 St. Olav’s University Hospital, Trondheim, Norway

**Keywords:** Alcohol consumption, Older adults, Elderly, Abstainers, China, Norway

## Abstract

**Background:**

There is little knowledge about the consumption of alcohol among Chinese and Norwegian older adults aged 65 years and over. The aim of this study was to investigate the prevalence and factors related to alcohol consumption among older adults in China and Norway.

**Methods:**

The Chinese Longitudinal Healthy Longevity Survey (CLHLS) data in 2008–2009 conducted in China and The Nord-Trøndelag Health Study data in 2006–2008 (HUNT3) conducted in Norway were used. Mulitvariable logistic regression was used to test the factors related to alcohol consumption.

**Results:**

The prevalence of participants who drink alcohol in the Chinese and Norwegian sample were 19.88% and 46.2%, respectively. The weighted prevalence of participants with consumption of alcohol in the Chinese sample of women and men were 7.20% and 34.14%, respectively. In the Norwegian sample, the prevalence of consumption of alcohol were 43.31% and 65.35% for women and men, respectively. Factors such as younger age, higher level of education, living in urban areas, living with spouse or partner, and better health status were related to higher likelihood of alcohol consumption among Norwegian older women and men; while reported better health status and poorer life satisfaction were related to higher likelihood of alcohol consumption among Chinese. In addition, rural males and older females with higher level of education were more likely to consume alcohol.

**Conclusion:**

The alcohol consumption patterns were quite different between China and Norway. Besides economic development levels and cultures in the two different countries, demographic characteristics, socioeconomic status, overall health status, and life satisfaction were associated with alcohol consumption as well.

## Background

Alcohol is a natural stimulant used in cultures across the globe, but the patterns of alcohol consumption differ across ethnic groups [1]. Cultural factors play an important role for drinking behavior [2], and prosperity is often associated with higher usage [3].

The consumption of alcohol is common among people aged 18 years or older in developed countries, such as Norway [4]. It is often linked to social events and special occasions, but also to everyday life such as relaxation at leisure time [5]. In Norway, individuals’ drinking patterns are quite stable regarding alcohol consumption per capita, increasing slightly from below 6 litres of pure alcohol per year in 1981 to 6.7 litres of pure alcohol per year in 2010 [6]. Alcohol consumption per capita (APC) is defined as the per capita amount of alcohol consumed in litres of pure alcohol in a given population [6]. In Norway, the intake of alcohol decreases with age in adult population [7–9]. The proportion of older people being abstinent from alcohol consumption is lower than before [7, 8, 10–12].

In China, considered to be a developing country, alcohol consumption is also a part of the culture. Alcohol consumption culture in China has been established for thousands of years [13, 14]. Chinese culture emphasizes social drinking, such as on the occasions of festivals, celebrations, and business meetings. Chinese culture has long promoted alcohol consumption, especially among men. Men who are capable of consuming a large quantity of alcohol are perceived as masculine [15, 16]. In addition, drinking is perceived to have the function of easing tension, relieving fatigue and facilitating social exchanges in social gatherings [16, 17]. China is undergoing rapid economic development and urbanization, and alcohol production and availability has increased over the past few decades; recorded alcohol consumption per capita rose from below 2 litres of pure alcohol per year in 1981 to 5.0 litres of pure alcohol per year in 2010 [6]. In China, the prevalence of alcohol consumption has been reported higher in younger age groups than in older groups [18, 19], and the prevalence of no consumption of alcohol increased with older age [18].

The consumption of alcohol is tied to regulations and rules which reflect each country’s alcohol policy [20] and the norms of society [20, 21]. There is no regulation forbidding alcohol consumption among adults in these two countries. China does not have enforceable legal regulations about drinking age, home-made alcohol beverages, and when or where alcoholic beverage can be sold [22]. However, there are several regulations covering these behaviors in Norway [23]. In addition, consumption of alcohol (versus no consumption) may be related to socioeconomic factors as well as to health status and life satisfaction. In Norway, adults in urban areas are more likely to be current drinkers, which means they have drunk in the recent 12 months, compared with those living in rural areas. Men are also more likely than women to be current drinkers [24, 25]. But in China, there are different findings about the relationship between rural/urban residency and the consumption of alcohol [18, 26]. In China, the consumption of alcohol among adults has previously been found more prevalent among those with lower level of education both for urban and rural areas [26]. In a more recent study, the prevalence of alcohol consumption was higher among adults with high education than among those with low education, but the analysis was not adjusted for confounders [18]. In Norway, alcohol consumption has been found to be more common in populations with higher education, in better economic status, and in older adults [27]. In a British study about alcohol consumption in older people, those who experienced a better-perceived health status and had a fairly active and sociable lifestyle were more likely to consume alcohol [28]. As far as we know, the knowledge about relationships between alcohol consumption and level of education, health status as well as life satisfaction is scarce in older adults in both China and Norway.

We know that older adults are at increased risk of harm from alcohol consumption due to physiological changes associated with aging, such as having a lower tolerance to alcohol, suffering from chronic illnesses, taking medications, or having functional impairments [29]. However, there are few studies about the consumption of alcohol in older adults in both countries [30, 31]. Exploring the prevalence of alcohol consumption and factors related to drinking among older adults is therefore desirable in providing significant knowledge for health care policy makers and professionals, so adequate steps toward a healthy aging can be taken in the years to come. There is a great need to conduct research in this area [12, 32]. In addition, alcohol consumption is related to culture and may differ across different levels of socioeconomic development. China and Norway have different cultural environments and levels of socioeconomic development. A comparative study of these two countries may provide better understanding of the impact of socioeconomic status and culture on alcohol consumption and help to identify potential modifiable factors about drinking.

The aim of the present study was to examine the prevalence and factors related to alcohol consumption among older adults in China and Norway.

## Methods

For this study, we performed a secondary data analysis using two large cross-sectional studies conducted in China and Norway at approximately the same time.

### Samples from each data set

*The Chinese Longitudinal Healthy Longevity Survey (CLHLS) in 2008–2009* selected participants from counties and cities in 22 of China’s 31 provinces. The population in the 22 provinces represents 85% of the total population of China. The study targeted institutionalized and community-living older adults. All centenarians from the selected areas who agreed to participate were included in the study. Based on gender and place of residence (i.e., living in the same street, village, city, or county) for a given centenarian, randomly selected octogenarians and nonagenarians were also sampled. This matched-recruitment procedure resulted in an over-sampling of the oldest old and older men. In the *CLHLS*, a weight of age–sex–urban/rural residence in the sample with the distribution of the total population in the sampled 22 provinces was employed to reflect the unique sampling design [33–35]. Older adults living in institutions were excluded in this analysis. In total, about 16,255 community dwelling residents aged ≥65 years were included. The unweighted mean age of the total sample was 87.4 (*SD* 11.4). The number of unweighted male participants was 6884 (42.4%), and the unweighted female participants was 9371 (57.6%).All participants in this study sample answered the main question of interest, consumption of alcohol.

*The Nord-Trøndelag Health Study in 2006–2008 (HUNT3) was conducted* over a study period of 2 years. The Nord-Trøndelag Health Study is one of the largest health studies ever performed in Norway. *HUNT3* was the third cross-sectional HUNT study which was completed between October 2006 and June 2008. Nord-Trøndelag is one of 19 counties in Norway. Nord-Trøndelag county had a population size of 128,694 in 2006. In many aspects, Nord-Trøndelag is considered fairly representative of Norway (geographically, and regarding economy, industry, sources of income, trends in work related disability, age distribution, morbidity and cause specific mortality) [36]. All adult residents (aged ≥20 years) in the Nord-Trøndelag County were asked to participate in the study. In all, 11,545 residents (5461 men) of 12,255 residents (5610 men) aged 65 and older participated and answered questions on alcohol consumption. In total, 816 did not answer the main question of interest, i.e. about alcohol consumption. Those not responding were more often women, living alone and having high age than those responding to the question (*p* < 0.01). Due to missing information on individual independent variables, the *N* varied from 11,545 to 9631 in this study. The mean age was 73.7 (*SD* 6.3) years, with an age range from 65 to 101 years. The rate of participation decreased with age from 71% among people aged 60–69 years to 18% among the oldest aged 90–99 years [37, 38]. The participants were individuals who could meet at an examination station. In this case, those with the most severe conditions and alcohol problems are underrepresented [39]. Older adults living in institutions were not included.

### Measures

The dependent variable was alcohol consumption. Each dataset contained questions directly pertaining to alcohol consumption as well as variables potentially related to consumption of alcohol (i.e. self-rated general health, life satisfaction and socio-demographic information).

*CLHLS* had a question about alcohol consumption: “Do you drink alcohol at present?” Consumption of alcohol was coded as 1 when the participant answered “drink alcohol at present”. *HUNT* 3 had a question about consumption of alcohol: “How often in the last 12 months did you drink alcohol?” Consumption of alcohol was coded as 1 when the participant answered about once a month or more (including 4–7 times a week, 2–3 times a week, about once a week, or 2–3 times a month).

Socio-demographic information such as age at the time of survey completion, gender, education level, marital status (living spouse or partner versus not), and residence (rural versus urban living) was collected in both studies. *CLHLS* had years of schooling as a continuous variable on education. *HUNT3* only had education as a categorical variable, not a continuous one. We recoded the years of schooling in *CLHLS* into a categorical variable in order to compare with *HUNT 3*. The coding was as following: Illiteracy = 0 years of schooling, elementary school and middle school = 1–9 years of schooling, high school = 10–12 years of schooling, and college and university = more than 12 years of schooling. *CLHLS* had illiterate participants, but *HUNT3* didn’t have such an education category. Each nation used their own definitions of rural and urban areas [40, 41]. Urban and rural areas in the Chinese study were self-reported by the participants according to a strictly enforced residential permit system in China [34]. Rural and urban areas in the Norwegian study were self-reported by the responders and described in previous studies [40, 42].

Perceptions of general health were assessed with one self-reported question. The Chinese overall health status variable, “How do you rate your health at present?”, had five response categories ranging from 1 (*very good*) to 5 (*very poor*). This item had been used in previous Chinese studies [34, 43]. The Norwegian general health item, “How is your health at the moment?”, had four response categories ranging from 1 (*very good*) to 4 (*very poor*). The item has been used in several Norwegian studies [40, 42]. We reversed the coding of overall health on both surveys so that a higher score reflected a better health status.

Life satisfaction was assessed with one item in each dataset. The Chinese item asked “How do you rate your life at present?”, and had five response categories that ranged from 1 (*very good*) to 5 (*very poor*). This item had been used in previous Chinese studies [44, 45]. The Norwegian item, “How do you think about your present life situation?”, had seven response categories that ranged from 1 (*extremely satisfied*) to 7 (*extremely dissatisfied*). This item has been used in several Norwegian studies, both in hospital samples and population-based studies [34, 38, 40, 46].We reversed the coding of this item on both surveys so that a higher score reflected better satisfaction with life.

### Statistical analyses

The consumption of alcohol in the Chinese and Norwegian samples of women and men was presented as percentages. Multivariable logistic regression was used to test the dependent variable (consumption of alcohol coded as 1 versus no consumption of alcohol coded as 0). The explanatory variables with possible relationship to the consumption of alcohol were gender, age, level of education, marital status (living or not living with spouse or partner), living in rural (versus urban) areas, self-rated overall health and life satisfaction. We conducted univariable logistic regression analyses first. We then included those variables that were significantly associated with the dependent variable (*P* < 0.05), and those of interest to this study in the multivariable logistic regression analyses. Odds ratios and 95% confidence intervals are reported. In *CLHLS*, there was a category of “unable to answer” for self-rated overall health and life satisfaction. We treated “unable to answer” as a missing value. Weights were applied to reflect the unique sampling design of the *CLHLS*. The analyses about the *CLHLS* and *HUNT3* samples were conducted using the programs of SAS 9.3 (SAS Institute, Inc., Cary, NC, USA), and SPSS version 22.0 (SPSS, Chicago, Ill, USA), respectively.

## Results

The prevalence of alcohol consumption for the Chinese and Norwegian samples were 19.88% (weighted) and 46.2%, respectively. The weighted prevalence of participants with consumption of alcohol in the Chinese sample were 7.20% and 34.14% for women and men, respectively. In the Norwegian sample, the prevalence of consumption of alcohol were 43.31% and 65.35% for women and men, respectively. The prevalence of consumption of alcohol by gender, socio-demographic conditions, perceived health, and life satisfaction for both samples are described in Table 1.

**Table 1:** The prevalence of consumption of alcohol in *CLHLS* and *HUNT3* samples

	women						men					
	China (weighted)			Norway			China (weighted)			Norway		
Variables	Total N	alcohol consumption		Total N	alcohol consumption		Total N	alcohol consumption		Total N	alcohol consumption	
		n	Prev. (%)		n	Prev. (%)		n	Prev. (%)		n	Prev. (%)
	8686	625	7.20	6084	2641	43.31	7723	2637	34.14	5461	3569	65.35
Age												
65–74	5127	322	6.28	3633	1875	51.61	5106	1855	36.32	3455	2490	72.07
75–84	2714	233	8.59	2054	667	32.47	2229	677	30.37	1756	976	55.58
85+	845	70	8.24	397	99	24.94	388	105	27.12	250	103	41.20
Achieved level of education in^a^												
1 Illiteracy	5444	373	6.84	0			1721	550	31.39	0		
2 Elementary school and middle school	2910	224	7.71	3504	1346	38.41	4965	1807	36.40	2122	1261	59.43
3 High school	229	11	4.95	1214	646	53.21	661	175	26.47	1836	1242	67.65
4 College and University	89	11	11.84	637	396	62.17	367	956	26.06	817	647	79.19
Living in^b^												
URBAN areas	3697	282	7.63	3712	1742	46.93	3268	941	28.79	3292	2242	68.10
RURAL areas	4989	343	6.87	2311	857	37.08	4456	1696	38.06	2115	1281	60.57
Marital status^c^												
No living spouse or partner	4728	334	7.06	2946	1038	35.23	1964	683	34.77	1308	787	60.17
Living spouse or partner	3958	291	7.35	3136	1603	51.12	5760	1954	33.93	4151	2780	66.97
Overall health status^d^												
Poor	1489	68	4.56	2452	914	37.28	1111	283	25.49	1899	1129	59.45
Life satisfaction^e^												
Poor	606	41	6.75	63	25	39.68	388	105	26.99	64	42	65.62

^a, b, c, d, e^Having missing value in the *CLHLS* and *HUNT3* study

Findings from logistic regression analyses on factors associated with alcohol consumption are shown in Tables 2 and 3. For Chinese women, those aged 75–84 years and aged 85+ were more likely to drink alcohol than those aged 65–74 (OR = 1.572, 95% CI = 1.298–1.904; OR = 1.564, 95% CI = 1.132–2.160, respectively); but for Chinese men and Norwegians (both women and men) those aged 75–84 years were less likely to consume alcohol than those aged 65–74 years (OR = 0.792, 95% CI = 0.709–0.885, OR = 0.569, 95% CI = 0.499–0.648, OR = 0.490, 95% CI = 0.428–0.560, respectively). Similar findings were found for the oldest old Chinese men, and the oldest old Norwegians aged 85+ (women and men), in comparison to those aged 65–74. The odds ratios were 0.689 (95% CI = 0.537–0.884), 0.534 (95% CI = 0.407–0.702), and 0.311 (95% CI = 0.231–0.420), respectively. For those who completed high school education or college and university education, Norwegian women and men were more likely to consume alcohol than those who completed elementary school and middle school. Chinese illiterate older women were less likely to drink than those with elementary school and middle school education. But for Chinese men, illiterate individuals and those with high school or college and university education were less likely to drink than those with elementary school and middle school education. For those living in rural areas, Chinese men were more likely to consume alcohol than those living in urban areas (OR = 1.471, 95% CI = 1.327–1.631), but Norwegian women and men were less likely to consume alcohol than those living in urban areas (OR = 0.728, 95% CI = 0.646–0.821, OR = 0.760, 95% CI = 0.667–0.865, respectively). For those living with spouse or partner, Norwegian women and men were more likely to consume alcohol compared with those without living with spouse or partner(OR = 1.510, 95% CI = 1.336–1.707, OR = 1.277, 95% CI = 1.097–1.487, respectively); however, no significant relationship was found between marital status and alcohol consumption among Chinese women and men. Those with better overall health were more likely to consume alcohol both in the Chinese and Norwegian sample, regardless of gender. Chinese women and men with better life satisfaction were less likely to consume alcohol compared to those with poor life satisfaction (OR = 0.889, 95% CI = 0.792–0.998, OR = 0.910, 95% CI = 0.849–0.974, respectively), but no similar associations were found among Norwegian women and men samples.

**Table 2:** Logistic regression of factors associated with alcohol consumption (versus no alcohol consumption) in *CLHLS* and *HUNT3* samples (women)

	China (weighted)						Norway					
	Univariable analyses			Multivariable analyses			Univariable analyses			Multivariable analyses		
Variables	OR	95% CI		OR	95% CI		OR	95% CI		OR	95% CI	
Age												
65–74	1	ref		1	ref		1	ref		1	ref	
75–84	1.356*	1.035	1.777	1.572*	1.298	1.904	0.451***	0.403	0.505	0.569***	0.499	0.648
85+	1.402*	1.175	1.672	1.564*	1.132	2.160	0.311***	0.246	0.395	0.534***	0.407	0.702
Achieved level of education in												
Illiteracy	0.879	0.740	1.044	0.804^*^	0.667	0.969				−	−	−
Elementary school and middle school	1	ref		1	ref		1	ref		1	ref	
High school	0.623	0.338	1.149	0.612	0.330	1.137	1.823***	1.598	2.080	1. 631***	1.418	1.876
College and University	1.608	0.833	3.104	1.570	0.807	3.055	2.634***	2.214	3.135	2.125***	1.767	2.554
Living in												
URBAN areas	1	ref		1	ref		1	ref		1	ref	
RURAL areas	0.885	0.751	1.043	0.954	0.802	1.136	0.667***	0.599	0.741	0.728***	0.646	0.821
Marital status												
No living spouse or partner	1	ref		1	ref		1	ref		1	ref	
Living spouse or partner	1.045	0.887	1.231	1.166	0.973	1.398	1.922***	1.734	2.131	1.510***	1.336	1.707
Overall health status	1.334***	1.217	1.463	1.399***	1.261	1.552	1.481***	1.363	1.610	1.271***	1.151	1.404
Life satisfaction	1.062	0.958	1.178	0.889*	0.792	0.998	1.088**	1.032	1.147	1.022	0.958	1.090

Notes. CI confidence interval, OR odds ratio. *p < .05. **p < .01. ***p < .001

**Table 3:** Logistic regression of factors associated with alcohol consumption (versus no alcohol consumption) in *CLHLS* and *HUNT3* samples (men)

	China (weighted)						Norway					
	Univariable analyses			Multivariable analyses			Univariable analyses			Multivariable analyses		
Variables	OR	95% CI		OR	95% CI		OR	95% CI		OR	95% CI	
Age												
65–74	1	ref		1	ref		1	ref		1	ref	
75–84	0.768*	0.691	0.855	0.792*	0.709	0.885	0.485***	0.430	0.547	0.490***	0.428	0.560
85+	0.655*	0.520	0.826	0.689*	0.537	0.884	0.272***	0.209	0.353	0.311***	0.231	0.420
Achieved level of education in												
Illiteracy	0.822*	0.731	0.923	0.811*	0.718	0.915				−	−	−
Elementary school and middle school	1	ref		1	ref		1	ref		1	ref	
High school	0.639*	0.524	0.755	0.698*	0.579	0.842	1.428***	1.253	1.627	1.296***	1.128	1.487
College and University	0.616*	0.484	0.783	0.732*	0.572	0.939	2.599***	2.149	3.142	2.084***	1.097	1.487
Living in												
URBAN areas	1	ref		1	ref		1	ref		1	ref	
RURAL areas	1.512***	1.372	1.666	1.471***	1.327	1.631	0.719***	0.642	0.806	0.760***	0.667	0.865
Marital status												
No living spouse or partner	1	ref		1	ref		1	ref		1	ref	
Living spouse or partner	0.966	0.867	1.076	0.931	0.830	1.043	1.342***	1.181	1.526	1.277**	1.097	1.487
Overall health status	1.215***	1.155	1.279	1.260***	1.189	1.335	1.354***	1.237	1.483	1.196**	1.070	1.336
Life satisfaction	0.997	0.940	1.058	0.910**	0.849	0.974	0.050	0.990	1.113	0.955	0.889	1.027

Notes. *CI* confidence interval, *OR* odds ratio. *p < .05. **p < .01. ***p < .001

## Discussion

This study examined the prevalence of alcohol consumption among older women and men in China and Norway, and factors (i.e., socio-demographic status, perceived overall health, and life satisfaction) associated with alcohol consumption in the population. The alcohol consumption patterns were quite different in the two countries.

The prevalence of alcohol consumption in Norway was higher than the prevalence in China both for older women and men. We assume that the differences between the prevalence of alcohol consumption in China and Norway may be partially explained by different levels of economic development between these two countries. According to the most recent WHO data, greater economic wealth was broadly associated with higher levels of alcohol consumption and lower abstention rates [3]. The alcohol consumption levels in developed countries such as WHO European Region and Region of the Americas were the highest across the globe, while the alcohol consumption level in China was lower than WHO European Region and Region of the Americas [6]. For the past 30 years, although China has been undergoing rapid economic development and urbanization, the alcohol consumption per capita in China is still lower than that of Norway’s [6]. Culture may also play an important role. In China, most alcohol consumption occurs at social events such as festivals, weddings, and business interactions. Cultural norms encourage social drinking and discourage solitary drinking [47]. In Norway and the western countries, consumption of alcohol is often not only linked to social events and special occasions, but also to everyday life like relaxation at leisure time [5].

Furthermore, we found that men had a higher prevalence of alcohol consumption than women in both countries. This finding is consistent with previous studies [6, 26, 48] and may be partially due to cultural values and norms in these countries [49]. In Chinese culture, there is a long history of alcohol consumption among men. Chinese men have more social interactions to drink than women. In rural areas of China, men are more likely to do the heavy labor than women; hence, men may drink to relieve physical fatigue [48]. We believe that culture may play a less prominent role in gender differences in Norway [49], both in rural and urban areas. In addition, purchasing power is strongly associated with alcohol consumption. In China, men have more economic power than women. Evidence suggest that the gender gap in socioeconomic status is much smaller in Norway than in China. Thus, it is expected that the proportion of older women with alcohol consumption is higher in Norway than in China.

Among participants aged 65 and over, older age was negatively associated with alcohol consumption in Chinese men and Norwegian men and women. It is consistent with the findings from studies conducted in United States, Denmark and United Kingdom [8, 9, 50]. The reason why people with older age decreased the incidence of drinking may be higher sensitivity to alcohol, suffering from chronic diseases, or using medication among older adults [29, 51]. But Chinese women aged 75–84 years and 85+ years had higher likelihood of alcohol consumption than those aged 65–74 years. One explanation for this may be that older Chinese women often believe that drinking has a good effect on health and lack of awareness of alcohol-related health problems, making them more likely to consumption alcohol [14] .

Similar to previous studies conducted in Norway, our analysis found that higher level of education was positively associated with alcohol consumption among Norwegian older women and men [52, 53]. Nordfjærn’s study showed that Norwegian individuals with high education had lower levels of alcohol abstinence (4%) than those with basic education (7%), and high education was also related to more consumption [52]. Brunborg’s study showed that income was weakly associated with risk of heavy drinking, but higher education was associated with greater risk of heavy drinking [53]. Education is normally covering the socio-economic status in older people in Nordic social democratic countries with a welfare system. The Norwegian people with higher education were more likely to have better socioeconomic status, able to afford alcohol, and more often consume alcohol in social events [15, 28, 54, 55]. For the Chinese sample, older women with higher level of education (e.g., elementary school and middle school education) had a higher likelihood of alcohol consumption compared to illiterate women. But for Chinese men, illiterate individuals and those with high school or college and university education were less likely to drink than those with elementary school and middle school education. The associations between education and alcohol consumption between China and Norway were different. It may be related to the different levels of education distribution among the two countries’ samples. The Norwegian sample had a higher level of education particularly in college and university level, but the Chinese sample had a lower level of education with a high percentage of illiteracy and a low percentage of college and university.

Living in rural areas was negatively related to alcohol consumption among Norwegian women and men. The difference in drinking patterns of Norwegian women and men between rural and urban areas was supported by other studies from developed countries [2, 56, 57]. The reasons may be a combination of different factors, such as socioeconomic status, disparate custom, and religious norms, which the present study did not explore. One reason is that people living in urban area of developed countries are more likely to have better socioeconomic status, so they can better afford alcohol. The fact that a higher proportion of people in rural areas have higher prevalence of abstinence of alcohol may be partially due to conservative religious beliefs [56]. However, the relationship between rural/urban living and alcohol drinking was reversed in Chinese men. Older Chinese men living in rural areas were more likely to be a drinker when compared to those living in urban areas. This phenomenon may be partially attributable to the different cultural and legal backgrounds and health system characteristics. In China, there is a lack of comprehensive public policy on alcohol consumption. The Government has few restrictions for people to produce or access alcohol [16]. Home-made or underground–brewed rice wines are commonly used in rural areas of China because they are more affordable than other industry-produced alcohol beverages [14]. The Chinese traditional customs, popular in rural areas, believe that moderate drinking can benefit people’s health. The Chinese Traditional Medicine theory holds the belief that “alcohol is the leader of all kinds of medicine, and can guide other medicines to the place of disease” [47, 58], so many rural residents put herbs in alcohol and drink the alcohol to treat some diseases and symptoms such as back and leg pain caused by rheumatism [14]. The disparities of health systems in rural and urban areas of China exacerbate this situation. The relative lack of medical care facilities and professionals in rural areas make it more difficult to get access to health care. Hence, the rural populations are more likely to rely on the Chinese Traditional Medicine belief and treat medical problems using alcoholic beverages by themselves. There was no significant difference of alcohol use between rural and urban areas among Chinese women. This finding may be most likely due to the small percentage of alcohol consumption among Chinese women, and thus we are not able to test the rural/urban differences due to a small sample.

Living with a spouse or partner was positively associated with using alcohol in Norwegian women and men, which was supported by the results of a previous German study [55]. But this association was not significant in China. It may be due to the different drinking cultures. Chinese people drink more frequently at social occasions, but not with their spouse or partner at home. However, alcohol consumption is a part of relaxation and leisure activity in Norway. People often drink and enjoy time together with their spouse or partner.

Our study found that better self-reported health status was positively associated with high alcohol consumption. This was consistent with previous studies [47, 48]. Health status may be an important factor in impacting older adults’ decisions whether to drink or not. Older adults with better overall health may think they can sustain alcohol use and choose to drink, while those with poor health statuses may choose not to use alcohol [55, 59]. Alternatively, older adults with better health statuses who continue to drink might be a result of healthy survival effect.

This study found that poorer life satisfaction was related to higher likelihood of alcohol consumption among Chinese women and men. This finding was supported by previous studies [60, 61]. However, this relationship was not significant among Norwegian women and men, which may indicate that the other factors included in the analysis (socioeconomic and health conditions) rather than satisfaction in life had greater importance.

The strengths of this study were the comparative design of the two datasets that enable us to examining the prevalence and factors associated with alcohol consumption among older adults in a developed country (Norway) and a developing country (China). The *CLHLS* Study conducted in China and the *HUNT3* Study conducted in Norway both had relatively large sample size and reliable results.

### Limitations

There were several limitations in the present analysis. The definitions of current drinking were slightly different in the two surveys. The variable about current drinking in the *CLHLS* study was a dichotomous variable, while the variable about current drinking in *HUNT3* was frequency of alcohol consumption. We had no information about the type of alcohol beverage, frequency of drinking, amount of alcohol intake, and alcohol-related disorders from the datasets, therefore we were not able to investigate the relationships between alcohol consumption and health consequences in China and Norway. Further studies focusing on drinking type, frequency, quantity, and alcohol-attributable disease and injury between developed and developing countries are needed. Older adults living in institutions were not included in *HUNT3. CLHLS* included both institutionalized and community-living older adults. In order to compare the prevalence of alcohol consumption in older adults in China and Norway, we excluded the institutionalized older adults in *CLHLS*, which might result in a selection bias. This study was conducted among community-dwelling older adults. The findings may not be generalizable to those living in institutions.

## Conclusions

This is the first comparative study reporting the prevalence and related factors of alcohol consumption among older adults in a developed country (Norway) and a developing country (China). We found that alcohol consumption patterns and factors related to alcohol consumption among older women and men were different between the two countries. The overall prevalence of alcohol consumption in Norway was higher than that in China. The prevalence of alcohol consumption among older men was higher than that among older women both in China and Norway. Younger age, higher education, living in urban areas, living with spouse or partner and better health status were related to higher likelihood of alcohol consumption among Norwegian older women and men. Living in rural areas, better health status and poorer life satisfaction were related to higher likelihood of alcohol consumption among Chinese older men. Older age, higher education, better health status and poorer life satisfaction were related to higher likelihood of alcohol consumption among Chinese older women. These findings suggest that policy makers and healthcare professionals need to have a better knowledge about culture-related factors in order to promote healthy aging, but also to take the demographic characteristics, socioeconomic status as well as economic development levels into account when considering alcohol consumption.

## Abbreviations

APC: Alcohol consumption per capita
*CLHLS*: The Chinese Longitudinal Healthy Longevity Survey
*HUNT3*: The Nord-Trøndelag Health Study in 2006–2008
OR: Odds Ratio
SD: Standard deviation
WHO: World Health Organization

## References

[1] Galvan FH, Caetano R (2003). Alcohol use and related problems among ethnic minorities in the United States. Alcohol Res Heal.

[2] Dawson DA, Grant BF, Chou SP, Pickering RP (1995). Subgroup Variation in U.S. Drinking Patterns: Results of the 1992 National Longitudinal Alcohol Epidemiologic Study. J. Subst. Abuse.

[3] World Health Organisation. WHO Regions Country Profiles. 2014;1–202.

[4] Skogen JC, Knudsen AK, Myrtveit SM, Sivertsen B (2014). Abstention, alcohol consumption, and common somatic symptoms: the Hordaland health study (HUSK). Int J Behav Med.

[5] KnutHeim T. Older and use of drugs - elderly “preventable”? [Internet]. forebygging.no. 2015 [cited 2016 Jan 8] Available from: http://www.forebygging.no/Artikler/2015/Eldre-og-bruk-av-rusmidler%2D-kan-eldre-forebygges/.

[6] World Health Organization. Global status report on alcohol and health 2014. Glob. status Rep. alcohol [Internet]. 2014;1–392. Available from: http://www.who.int/substance_abuse/publications/global_alcohol_report/msbgsruprofiles.pdf.

[7] Moos RH, Brennan PL, Schutte KK, Moos BS (2004). High-risk alcohol consumption and late-life alcohol use problems. Am J Public Health.

[8] Moore A a, Gould R, Reuben DB, Greendale GA, Carter MK, Zhou K (2005). Longitudinal patterns and predictors of alcohol consumption in the United States. Am. J. Public Health.

[9] Bjørk C, Thygesen LC, Vinther-Larsen M, Grønbæk MN (2008). Time trends in heavy drinking among middle-aged and older adults in Denmark. Alcohol Clin Exp Res.

[10] Strandberg TE, Strandberg AY, Salomaa VV, Pitkälä K, Tilvis RS, Miettinen TA (2007). Alcoholic beverage preference, 29-year mortality, and quality of life in men in old age. J. Gerontol. A. Biol. Sci. Med Sci.

[11] Aira M, Hartikainen S, Sulkava R (2005). Community prevalence of alcohol use and concomitant use of medication--a source of possible risk in the elderly aged 75 and older. Int. J. Geriatr. Psychiatry..

[12] Støver M, Bratberg G, Nordfjørn T, Krokstad S. Use of alcohol and prescription drugs among elderly (60+) in Norway. The HUNT study, Norway. Use alcohol Prescr. drugs among Elder. Norway. HUNT study, Norw. HUNT forskningssenter, NTNU; 2012. p. 34.

[13] Alcohol GM, Markets E (1998). October.

[14] Hao W, Su Z, Liu B, Zhang K, Yang H, Chen S (2004). Drinking and Drinking Patterns and Health Status in the General Population of Five Areas of China.

[15] Zhang J, Wang J, Lu Y, Qiu X, Fang Y (2004). Alcohol abuse in a metropolitan city in China: a study of the prevalence and risk factors. Addiction.

[16] Hao W (1995). Alcohol policy and the public good: a Chinese view. Addiction.

[17] Lin TY, Lin DT (1982). Alcoholism among the Chinese: further observations of a low-risk population. Cult Med Psychiatry.

[18] Millwood IY, Li L, Smith M, Guo Y, Yang L, Bian Z (2013). Alcohol consumption in 0.5 million people from 10 diverse regions of china: Prevalence, patterns and socio-demographic and health-related correlates. Int. J. Epidemiol.

[19] Sun W, Schooling CM, Chan WM, Ho KS, Lam TH, Leung GM (2009). Moderate alcohol use, health status, and mortality in a prospective Chinese elderly cohort. Ann Epidemiol.

[20] Nordlund S. pular norms, alcohol policy and drinking behavior. In: Anderson P, Braddick F, Reynolds J, Gual A, editors. Alcohol Policy Eur. Evid. from Amphor. 2012. p. 24–31.

[21] Kumar BN (2008). Grøtvedt L.

[22] Tang Y, Xiang X, Wang X, Cubells JF, Babor TF, Hao W. WHO | Alcohol and alcohol-related harm in China: policy changes needed. Bull. World Health Organ. [Internet]. World Health Organization; 2013 [cited 2016 Jan 11];91:270–276 Available from: http://www.who.int/bulletin/volumes/91/4/12-107318/en/. 10.2471/BLT.12.107318 PMC3629448 23599550

[23] Karlsson T, Österberg E. Alcohol Policies in EU Member States and Norway [Internet]. [cited 2016 Mar 8] Available from: http://ec.europa.eu/health/ph_projects/1998/promotion/fp_promotion_1998_a01_27_en.pdf.

[24] Skogen JC, Knudsen AK, Mykletun A, Nesvåg S, Overland S. Alcohol consumption, problem drinking, abstention and disability pension award. The Nord-Trøndelag health study (HUNT). Addiction. 2012:98–108. 10.1111/j.1360-0443.2011.03551.x 21707810

[25] Nordlund S (1996). The use of alcohol and other drugs in Norway. Nor J Epidemiol.

[26] Wu B, Mao Z-F, Rockett IRH, Yue Y (2008). Socioeconomic status and alcohol use among urban and rural residents in China. Subst Use Misuse.

[27] Slagvold B, Løset KA. Alcohol consumption in old age. Trends and causes. What does the NorLAG.study tell? Forebygging.no. 2014.

[28] Hajat S, Haines A, Bulpitt C, Fletcher A (2004). Patterns and determinants of alcohol consumption in people aged 75 years and older: results from the MRC trial of assessment and management of older people in the community. Age Ageing.

[29] NIH_National_Institute_on_Aging. Alcohol Use In Older People [Internet]. 2015 [cited 2016 Jan 1]. Available from: https://www.nia.nih.gov/health/publication/alcohol-use-older-people.

[30] Frydenlund R. Older persons, alcohol and use of psychofarmaca. Review of existing literature. In Norwegian. Kompetansesentret-rus Oslo, Velferdsetaten Oslo kommune; 2012.

[31] Woo J, Ho SC, Yu ALM (2002). Lifestyle factors and health outcomes in elderly Hong Kong Chinese aged 70 years and over. Gerontology.

[32] Barry LC, Murphy TE, Gill TM (2011). Depression and functional recovery after a disabling hospitalization in older persons. J Am Geriatr Soc.

[33] Zeng Y, Liu Y, Zhang C, Xiao Z. Data quality assessment of the Chinese Longitudinal Healthy Longevity Survey 1998, 2000, and 2002 waves. Anal. Determ. Heal. Longev. Beijing: Peking University Press; 2004. p. 3–22.

[34] Gu D, Dupre ME, Liu G (2007). Characteristics of the institutionalized and community-residing oldest-old in China. Soc Sci Med.

[35] Zeng Y, Dudley L (2008). Poston J.

[36] Krokstad S, Westin S (2002). Health inequalities by socioeconomic status among men in the Nord-Trøndelag health study. Norway Scand J Public Health.

[37] Krokstad S, Langhammer A, Hveem K, Holmen TL, Midthjell K, Stene TR (2013). Cohort profile: the HUNT study. Norway Int J Epidemiol.

[38] Holmen J, Midthjell K, Krüger Ø, Langhammer A, Holmen TL, Bratberg GH (2003). The Nord-Trøndelag health study 1995-97 (HUNT 2): objectives, contents, methods and participation. Nor Epidemiol.

[39] Langhammer A, Krokstad S, Romundstad P, Heggland J, Holmen J. The HUNT Study: participation is associated with survival and depends on socioeconomic status, diseases and symptoms. BMC med. Res. Methodol. [internet]. BMC medical research methodology; 2012;12:143. Available from: BMC Medical Research Methodology. 10.1186/1471-2288-12-143 PMC3512497 22978749

[40] Holmen J et al. The Nord-Trøndelag Health Survey 1984–86. Purpose, background and methods. Participation, non-participation and frequency distributions. Health service research. Center for samfunnsmedisinsk research; 1990.

[41] Kamal-chaoui L, Leman E, Rufei Z. Urban Trends and Policy in China. OECD Reg. Dev. Work. Pap. 2009;2009/1:OECD Publishing.

[42] HUNT. The health Study in Nord-Trøndelag (HUNT) [Internet]. 2009 [cited 2009 Jan 20] Available from: https://www.ntnu.edu/hunt.

[43] Peng R, Wu B (2015). Changes of health status and institutionalization among older adults in China. J Aging Health.

[44] Zhang W, Liu G (2007). Childlessness, psychological well-being, and life satisfaction among the elderly in China. J Cross Cult Gerontol.

[45] Zhu H. Unmet Needs in Long-term Care and their Associated Factors Among the Oldest Old in China. BMC Geriatr. ???; 2015;15:1–11. 10.1186/s12877-015-0045-9 PMC4408585 25880545

[46] Helvik A-S, Jacobsen GW, Hallberg LR-M. Effects of Impaired Hearing on Perceived Health and Life Situation. Scand. J. Disabil. Res Taylor & Francis Group; 2006;8:263–277.

[47] Hao W, Young D, Xiao S, Li L, Zhang Y (1999). Alcohol consumption and alcohol-related problems: Chinese experience from six area samples, 1994. Addiction.

[48] Zhou X, Su Z, Deng H, Xiang X, Chen H, Hao W (2006). A comparative survey on alcohol and tobacco use in urban and rural populations in the Huaihua District of Hunan Province. China Alcohol.

[49] Bratberg GH, C Wilsnack S, Wilsnack R, Håvås Haugland S, Krokstad S, Sund ER, et al. Gender differences and gender convergence in alcohol use over the past three decades (1984–2008), The HUNT Study, Norway. BMC Public Health [Internet]. BMC Public Health; 2016;16:723. Available from: http://bmcpublichealth.biomedcentral.com/articles/10.1186/s12889-016-3384-3. 10.1186/s12889-016-3384-3 PMC4974746 27492155

[50] Merrick EL, Horgan CM, Hodgkin D, Garnick DW, Houghton SF, Panas L (2008). Unhealthy drinking patterns in older adults: prevalence and associated characteristics. J Am Geriatr Soc.

[51] NIHSeniorHealth. Alcohol Use and Older Adults - Alcohol and Aging [Internet]. 2015 [cited 2016 Jan 1] Available from: http://nihseniorhealth.gov/alcoholuse/alcoholandaging/01.html.

[52] Nordfjærn T, Brunborg GS. Associations Between Human Values and Alcohol Consumption Among Norwegians in the Second Half of Life. Subst. Use Misuse [Internet]. 2015;50:1284–93. Available from: 998237. 10.3109/10826084.2014.998237 25603494

[53] Brunborg GS. Positive and negative affectivity as risk factors for heavy drinking in the second half of life: a prospective cohort study. Addiction. 2016;142 10.1111/add.13718 27935667

[54] Rao R, Schofield P, Ashworth M (2015). Alcohol use, socioeconomic deprivation and ethnicity in older people. BMJ Open.

[55] Weyerer S, Schäufele M, Eifflaender-Gorfer S, Köhler L, Maier W, Haller F (2009). At-risk alcohol drinking in primary care patients aged 75 years and older. Int J Geriatr Psychiatry.

[56] Booth BM, Curran GM (2006). Variations in drinking patterns in the rural south: joint effects of race, gender, and rural residence. Am J Drug Alcohol Abuse.

[57] Borders TF, Booth BM (2007). Rural, suburban, and urban variations in alcohol consumption in the United States: findings from the National Epidemiologic Survey on alcohol and related conditions. J Rural Health.

[58] Cochrane J, Chen H, Conigrave MK, Hao W (2003). Alcohol use in China. Alcohol Alcohol.

[59] Borok J, Galier P, Dinolfo M, Welgreen S, Hoffing M, Davis JW (2013). Why do older unhealthy drinkers decide to make changes or not in their alcohol consumption? Data from the healthy living as you age study. J Am Geriatr Soc.

[60] Fergusson DM, McLeod GFH, Horwood LJ, Swain NR, Chapple S, Poulton R (2015). Life satisfaction and mental health problems (18 to 35 years). Psychol Med.

[61] Liu J (2015). Research on the Elderly’s life satisfaction and the influence factors-analysis based on National Baseline Data of China health and retirement longitudinal study (CHARLS). Sci Res Aging.

